# Revisiting the Regenerative Therapeutic Advances Towards Erectile Dysfunction

**DOI:** 10.3390/cells9051250

**Published:** 2020-05-19

**Authors:** Ming-Che Liu, Meng-Lin Chang, Ya-Chun Wang, Wei-Hung Chen, Chien-Chih Wu, Shauh-Der Yeh

**Affiliations:** 1 Department of Urology, Taipei Medical University Hospital, Taipei 11031, Taiwan; d204097002@tmu.edu.tw (M.-C.L.); ccwu@h.tmu.edu.tw (C.-C.W.); 2 Clinical Research Center, Taipei Medical University Hospital, Taipei 11031, Taiwan; 3 Graduate Institute of Clinical Medicine, school of Medicine, College of Medicine, Taipei Medical University, Taipei 11031, Taiwan; 4 School of Dental Technology, College of Oral Medicine, Taipei Medical University, Taipei 11031, Taiwan; 5 Department of Urology, Fu Jen Catholic University Hospital, Fu Jen Catholic University, New Taipei City 242, Taiwan; oro.tidyscoundrel@gmail.com; 6 School of Medicine, College of Medicine, Fu Jen Catholic University, New Taipei City 242, Taiwan; 7 Graduate Institute of Applied Science and Engineering, Fu Jen Catholic University, New Taipei City 242, Taiwan; 8 TCM Biotech International Corp., New Taipei City 22175, Taiwan; yachunwang@tcmbio.com (Y.-C.W.); howard@tcmbio.com (W.-H.C.); 9 Department of Education and Humanities in Medicine, School of Medicine, College of Medicine, Taipei Medical University, Taipei 11031, Taiwan; 10 Department of Urology and Oncology, Taipei Medical University Hospital, Taipei 11031, Taiwan; 11 Department of Urology, School of Medicine, College of Medicine, Taipei Medical University, Taipei 11031, Taiwan

**Keywords:** erectile dysfunction (ED), platelet-rich plasma (PRP), platelet-derived biomaterials, stem cells, regenerative therapy, intracavernosal injection, intracavernosal pressure (ICP)

## Abstract

Erectile dysfunction (ED) is an inability to attain or maintain adequate penile erection for successful vaginal intercourse, leading to sexual and relationship dissatisfaction. To combat ED, various surgical and non-surgical approaches have been developed in the past to restore erectile functions. These therapeutic interventions exhibit significant impact in providing relief to patients; however, due to their associated adverse effects and lack of long-term efficacy, newer modalities such as regenerative therapeutics have gained attention due to their safe and prolonged efficacy. Stem cells and platelet-derived biomaterials contained in platelet-rich plasma (PRP) are thriving as some of the major therapeutic regenerative agents. In recent years, various preclinical and clinical studies have evaluated the individual, as well as combined of stem cells and PRP to restore erectile function. Being rich in growth factors, chemokines, and angiogenic factors, both stem cells and PRP play a crucial role in regenerating nerve cells, myelination of axons, homing and migration of progenitor cells, and anti-fibrosis and anti-apoptosis of damaged cavernous nerve in corporal tissues. Further, platelet-derived biomaterials have been proven to be a biological supplement for enhancing the proliferative and differentiation potential of stem cells towards neurogenic fate. Therefore, this article comprehensively analyzes the progresses of these regenerative therapies for ED.

## 1. Introduction

**Figure 1:**
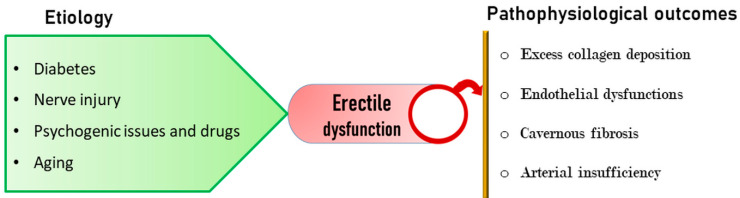
Pathophysiology of erectile dysfunction (ED). Aging, psychogenic issues, drugs, nerve injury, and diabetes represent major etiological factors leading to physiological changes such as excess collagen deposition, endothelial dysfunction, cavernous fibrosis, arterial insufficiency in penile corpus cavernosum, and loss of erectile function.

Erectile dysfunction (ED) severely impacts the personal, social, and sexual life of patients [1,2] and is found be more frequent among the middle aged and aging population [3]. In the context of sexual health, ED affects all domains such as desire, arousal, erectile function, and ejaculation/orgasm [4]. Therefore, its early diagnosis and management are highly imperative. The etiology of ED may include aging, psychological disorders, spinal injury/nervous disorders, diabetes, sleep apnea, chronic obstructive pulmonary disease, renal insufficiency, cavernous fibrosis, Peyronie’s disease, and the adverse effect of drugs (Figure 1) [3]. In addition, excessive use of drugs and over stress also lead to progressive ED. The progression of diabetes, cardiovascular disorders, and hypertension also pose a high risk of ED and hence decreased libido.

To address therapeutic alternatives, both traditional and current pharmacologic approaches have been explored. The traditional measures including use of animal, insect, arthropod, and herbal products, exercise, and acupuncture mainly addresses the balance between physical and emotional response along with hormone regulation [5,6]. The slow recovery through lifestyle changes as the first step to overcome ED has prompted the scientific community to explore other treatment measures such as available surgical/non-surgical intervention (drugs) for rapid recovery [3]. Phosphodiesterase 5 (PDE5) inhibitors such as sildenafil citrate, vardenafil, and tadalafil are the most popular choice of drugs [7]. Moreover, the use of PDE5 drugs may cause headache, nasal congestion, and dyspepsia [8]. Besides, the sublingual apomorphine is another recently developed alternative drug for ED [8]. In the case of no response to these oral medications, the intracavernous injections of alprostadil, papaverine, and phentolamine have proven to be successful alternatives. However, owing to their short-term effect, these interventions do not fully cure ED. The use of vacuum constriction and penile prostheses is considered as a last resort mostly in the case of older patients with much less frequency of sexual encounters [7]. The high cost, invasiveness, and irreversibility are limiting factors for surgical intervention for ED.

However, the recent development in regenerative medicine has directed the route to develop cell-based therapy for the feasibility of long-term treatment of ED. Stem cells and platelet-rich plasma (PRP) are widely studied candidates for regenerative therapies for various disorders [9]. Based on this fact, we discuss below the various basic research and clinical studies on the therapeutic potential of stem cells, PRP, and their combination with stimulatory agents for regenerating damaged cavernous nerve in penile corporal tissues.

## 2. Current Therapeutic Approaches for Erectile Dysfunction 

**Figure 2:**
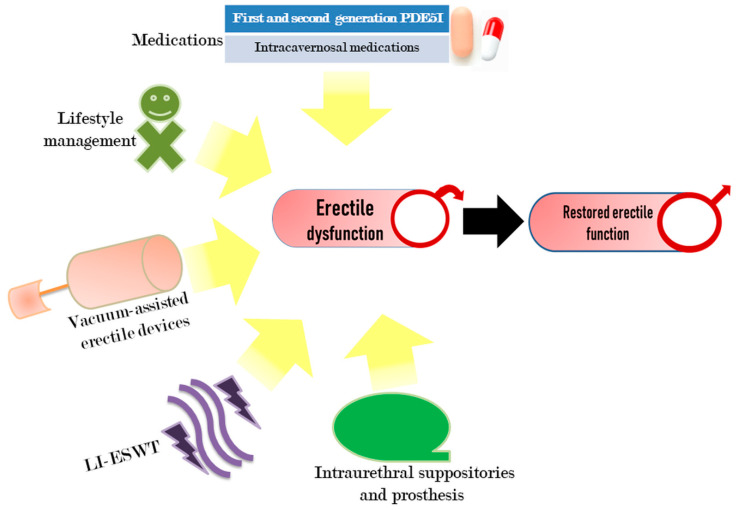
Current surgical and non-surgical practices for ED treatment. The most commonly available therapeutic interventions for ED include PDE5I and other intracavernosal drugs, lifestyle management, vacuum-assisted erectile devices, low-intensity extracorporeal shockwave therapy (LI-ESWT), intraurethral suppositories, and prosthesis.

A complete recovery through therapeutic interventions of ED is still a challenging task in imparting erectile functions, which is necessary to restore personal and social confidence. The currently available most common treatment measures include lifestyle management, oral drugs, psychotherapy, vacuum-assisted devices, shock wave therapy, and injectable suppositories (Figure 2).

### 2.1. Lifestyle Modifications and Oral Medications for Addressing ED

Lifestyle-associated changes remain the first step in improving ED. Specifically, reduced smoking and alcohol consumption along with moderate exercise and choice of diet seem efficacious [4]. It has also been evidenced that suppressed pathological conditions such as diabetes, cardiovascular disease, hypertension, and psychological disorders through modifying lifestyle are also likely to reduce the threat of ED [10]. Moreover, the eroticization of ED aids changes the focus from a perceived disability of the patient to sexual pleasure imparted by the partner, thereby improving both erectile aid compliance and effectiveness [11]. Thus, lifestyle-related interventions not only reduce the risk of ED, but also help to restore and improve sexual performance.

Further, the class of novel agents known as type-5 phosphodiesterase (PDE5) inhibitors has been proven to be efficacious in managing ED. These drugs include sildenafil, vardenafil, tadalafil, and avanafil. The generation of cGMP and its phosphodiesterase type 5 (PDE5)-mediated breakdown complete the erection cycle [10], and commonly used oral medications target this erection metabolic pathway to cGMP concentration, resulting in improved erectile function. However; these drugs are only functional when the patient has a nerve system to responsive stimulus [1,10]. These drugs should be prohibited during treatment of chest pain, and precaution should be taken when given with alpha receptor blocking drugs and CYP3A inhibitors such as azole antifungals, antiretroviral protease inhibitors, or marcolide antibiotics [12]. Among the second generation of PDE5I, avanafil is commonly preferred among physicians due to the greater efficacy and safety compared to first-generation PDE5I drugs. The reported common side effects associated with PDE5I are headache, dyspepsia, dizziness, flushing, nasal congestion, rhinitis, altered vision, back pain, and myalgia.

### 2.2. Intracavernosal Medications and Intraurethral Suppositories

Medications such as prostaglandin E1 (PGE1) muscle relaxant and erectile stimulant standalone or in combination with papaverine, phentolamine, and vasoactive intestinal peptide (VIP) are reasonable first-line ED therapies, which are administered through penile corpora [4,10,13]. This therapy is considered as an alternative among those non-respondent to PDE5I treatment [14]. The intracavernosal injection exhibits a higher discontinuation rate due to the associated pain; however, TriMix (a combination of PGE1, papaverine, and phentolamine) treatment seems to be less painful [15]. Further, compared to the intraurethral mode, the intracavernosal administration of alprostadil, an analogue of PGE1, has been demonstrated to be more effective in restoring penile blood flow and erectile functions [16], and the side effects such as penile and urethral pain along with risk of urethral infections reduce the acceptance of therapy [17].

### 2.3. Shockwave Therapy 

**Figure 3:**
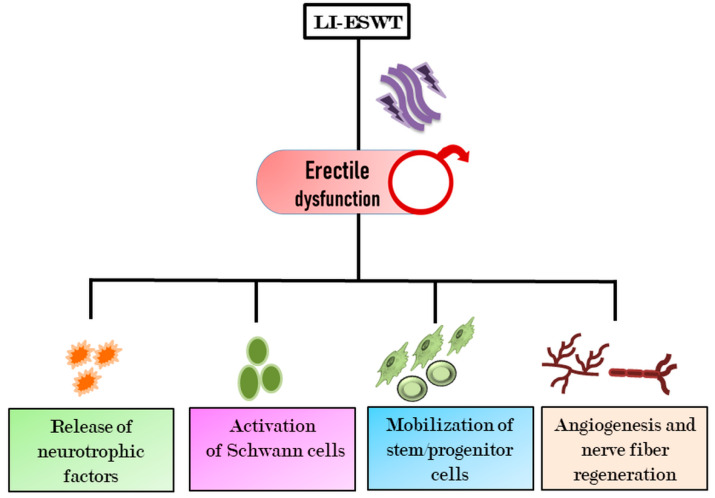
Impact of low-intensity extracorporeal shockwave therapy (LI-ESWT)-mediated therapeutic activities in ED. During the process of ED recovery, LI-ESWT increases the homing of stem/progenitor cells, activates Schwan cells, releases neurotrophic factors, and triggers angiogenesis, thereby improving erectile function.

The therapeutic efficacy of shockwaves (SWs) is attributed to their ability to carry energy and propagate through tissue. Though the underlying mechanism of action of SWs on corpora cavernosa remains to be understood, it is anticipated that the acoustic pressure-generated shock could compress targeted tissues, followed by expansion, which takes place over the tissue’s tensile elements. To improve vasculogenic ED, low-intensity extracorporeal shockwave therapy (LI-ESWT) has been reported to be a promising non-invasive candidate to trigger revascularization and restoring blood vessel functions (Figure 3) [18,19,20]. During this therapeutic process, LI-ESWT activates the release of angiogenic factors to promote neovascularization and improve hemodynamics in injured penile tissue without any adverse impact [21,22]. LI-ESWT also improves response to PDE5I drugs among PDE5I non-respondent patients, resulting in improved erectile functions [23]. The angiogenic stimulation properties of LI-ESWT have been well explored in developing regenerative therapy for chronic wounds, peripheral neuropathy, and cardiac disorders [21], which may be mediated by the release of angiogenic factors such as eNOS and VEGF along with other growth factors [24,25]. In the hind limb ischemia rat model, VEGF has been shown to serve as a homing factor for circulating progenitor cells (CPCs) [26]. In diabetic ED rats, LI-ESWT seems to downregulate the receptor of advanced glycation end products (RAGE) and upregulate factors such VEGF, nNOS, and α-SMA, leading to increased content of smooth muscles, endothelial cells, and ECM [27]. In a rat model of pelvic neurovascular injury (PNVI)-induced ED, LI-ESWT ameliorates impaired penile hemodynamics by improving nerve cell content with an increase in nNOS-positive nerves, Schwann cells, and axons in dorsal penis [28].

In an interesting study on diabetes mellitus-induced ED rats, the ESWT with modified Ojayeonjonghwan (Korean herbal formula, KH-204) synergistically improved erectile function through enhancing intracavernosal pressure (ICP) and restoring smooth muscle contents and other potency-associated parameters such as vascular endothelial growth factor, neuronal nitric oxide synthase (NOS), endothelial NOS, and platelet endothelial cell adhesion molecule-1 [29]. Contrary to the above-reported studies, no significant effect of LI-ESWT on nerve regeneration in ED pathophysiology has also been documented [30]. Even though LI-ESWT facilitates the recruitment and homing of stem/progenitor cells [31,32], the reduced population of these cells in older rats revealed suppressed efficacy of this intervention. Based on the above-mentioned evidence, though LI-ESWT is considered as a promising approach for ED, more clinical studies and standardization of the procedure are required for its approval and acceptance.

## 3. Emerging Regenerative Therapeutic Approaches for ED

### 3.1. Improving Erectile Functions Through Stem Cell-Based Bioengineering

**Figure 4:**
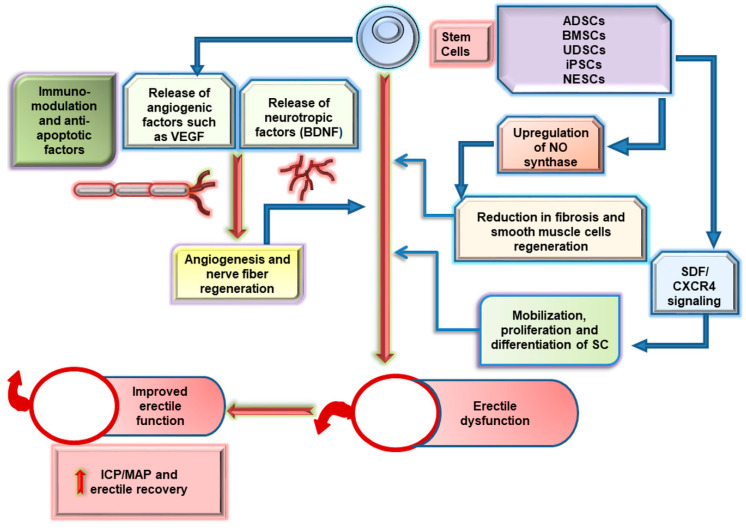
Mechanistic insight into stem cell-based therapy of ED. The most widely studied stem cell types for ED include adipose-derived stem cells (ADSCs), bone marrow-derived stem cells (BMSCs), urine-derived stem cells (UDSCs), induced pluripotent stem cells (iPSCs), and neural embryonic stem cells (NESCs). During repair and regeneration, the stem cells regulate signaling pathways such as SDF/CXCR4 to mobilize, proliferate, and differentiate stem cells into nerve and endothelial cells. Further, these cells also release growth factors such as VEGF and BDNF to promote angiogenesis and nerve fiber regeneration, respectively. Upregulation of nitric oxide (NO) synthase results in reduced fibrosis and an increase in smooth muscle cell content in corpus cavernosum. Eventually, the immunomodulatory and anti-apoptotic impact of stem cells controls any further injury to penile nerves or muscles. Thus, the cumulative effect of stem cells renders it a potent regenerative therapeutic candidate for ED. MAP, mean arterial pressure.

Recently, stem cell therapy has emerged as a promising regenerative option due to their ability of proliferation and multi-differentiation into specific cells to repair damaged tissues (Figure 4). The steps in stem cell therapy involve their isolation, sub-culturing, and proliferation, followed by sorting, and finally, the delivery at injured cavernous nerve and post-treatment assessments [33].

During repair and regeneration, stem cells secrete exosomes, cytokines, and growth and neurotropic factors and therefore play a crucial role in restoring major pelvic ganglion (MPG). Of them, the autologous MSCs are effective at providing regenerative therapies for various disorders without any significant adverse events [34]. These MSCs exhibit reduced risk of tissue rejection; hence remaining free from ethical and regulatory prospective concerns [35]. Additionally, the use of autologous stem cells is economical, and no cell line development is needed for their clinical applications [36]; however, it is difficult to harvest sufficient autologous MSCs from patients with diabetes and autoimmune disorders and older and underweight persons [37], and this is not recommended for persons with genetic defects or mutations [36]. Notably, the allogenic stem cells are more potent for large-scale use due to their availability, homogeneity, prior characterization, and absence of patient biopsy procedures; nonetheless, the stability of allogenic stem cells is a considerable challenge for clinical applications. In the coming sections, we specifically describe the studies investigating the impact of various types of stem cells on cavernous nerve injury (CNI)-induced ED in a rat model.

### 3.2. Potential Rescuing Effect of Adipose-Derived Stem Cells on ED

The majority of studies have employed ADSCs alone or combined them with other cells/growth factors [38,39]. During repair and regeneration, stem cells differentiate into smooth muscle cells (SMCs), vascular endothelial cells, and peripheral nerve cells, leading to a rescuing effect on CNI [40]. The improvement of erectile function among diabetic animals has been achieved by reducing apoptotic cells, increasing the contents of smooth muscle and endothelium with significantly enhanced expression of endothelial nitric oxide synthase and neuronal nitric oxide (NO) synthase, the ratio of smooth muscle to collagen, as well as secretion of VEGF [41]. In a rat model of diabetes-induced ED, it was demonstrated that compared to individual administration, the combined ADSCs and LI-ESWT exhibited a better therapeutic effect [42]. Further, though no positive interaction between ADSC therapy and LI-ESWT was revealed, both were able to be increase the expression of α-smooth muscle cells, nNOS, and Von Willebrand factor in corpus cavernosum. Additionally, cells also secrete bioactive molecules, which regulate cellular pathways such as angiogenesis, immuno-modulation, inflammatory response, anti-apoptotic activities, revascularization, self-renewal, proliferation, and differentiation of stem cells, leading to tissue regeneration [43]. The homing of MSCs to injured tissues occurs through the release of chemokines and their interaction with the stem cells’ respective receptors such as CCR1-10, CXCR1-2, CX3CR1, and CXCR4-6 for their CC, CXC, CX3C, and C chemokines, respectively [43]. These interactions are influenced by cell source and their passage and culture conditions. During injury, the expression of SDF-1 increases, resulting in a higher flux of stem cells, which further improves the retention/homing of MSCs at the wound site [44]. The interaction between the SDF-1-CXCR4 receptor also remains elevated in the presence of cytokines such as INF-α and TNF-α [45], which boost the therapeutic efficacy of MSCs [44,46]. Reports have evidenced that ADSCs and ADSC-derived lysate could improve ICP, reduce fibrosis, and increase nitric oxide synthase (nNOS) in penile nerve along with an increase in smooth muscle cells (SMC) and collagen, resulting in restored erectile activity in rats [47]. This therapeutic efficacy implies the paracrine, neurogenic, and anti-apoptotic roles of ADSCs. Moreover, compared to perineural mode, the intracavernously-administered ADSCs seem to be more effective in erectile recovery by enhanced SDF-1 levels in major pelvic ganglia (MPG), thus localizing ADSCs to MPG, leading to regenerated SMC and collagen in corpus cavernosa [48]. The intracavernously-administered ADSCs have also been demonstrated to be induced into neural-like cells, resulting in an increased maximal ICP, ratio of maximal ICP to mean arterial pressure, the number of myelinated axons and neuronal nitric oxide synthase-positive fibers in dorsal penile nerve, and the ratio of smooth muscle to collagen in a bilateral nerve crush injury rat model [49]. Similarly, other studies have also attempted to evaluate the pre-clinical efficacy of ADSCs to overcome ED [50]; however, more extensive studies are required to establish its clinical role. To further enhance the potency of ADSCs, various synergistic approaches have been carried out. In a seminal study, Lee et al. reported that the entrapped ADSCs within poly-lactic-co-glycolic acid (PLGA) along with brain-derived neurotropic factor (BDNF) and basic fibroblast growth factor (bFGF) showed considerably increased nNOS, cGMP, SMC, and collagen, resulting in the restoration of the ICP/mean arterial pressure (MAP) ratio; indicating restored erectile activity in a rat model of post-prostatectomy ED [51]. In a similar trend, ADSCs and BDNF immobilized poly-lactic-co-glycolic (PLGA) membrane significantly improved phosphor-eNOS expression and smooth muscle/collagen content [52]. Interestingly, similar to intracavernously-injected human ADSCs, the subcutaneous penile injection of bFGF hydrogel has also been shown to inhibit smooth muscle atrophy and improve ICP in corpus cavernosum and recovered erectile function among CNI rats [53]. In a seminal study, the implanted autologous ADSCs seeded onto allogenic adipose matrix repaired the injured CN and substantially restored erectile function in rats [54]. The use of pharmacologic interventions such as udenafil drug in combination with BDNF and ADSCs increased VEGF expression, angiogenesis, and protection of cavernous nerve in CNI [55]. A recombinant DNA technology based study by Yang et al. revealed that intracavernously-injected ADSCs infected with lenti-rBDNF increased nNOS and smooth muscle in penile tissues of a rat model of cavernous nerve injury, effectively improving erectile dysfunction caused by cavernous nerve injury [56]. To further improve the therapeutic efficacy, co-overexpression of VEGF and GDNF in ADSCs has also been attempted in a neurogenic ED rat model, which showed promoted cavernous nerve repair, inhibited penile fibrosis, and preserved vascular endothelium [57]. Magnetization of ADSCs with NanoShuttle magnetic nanoparticles could also retain them in corpus cavernosum and enhance the ICP/MAP, alpha smooth muscle actin (α-SMA), and platelet endothelial cell adhesion molecule-1, leading to improved erectile function [58]. Furthermore, using an assistive magnetic field, even low doses of NanoShuttle-bound ADSCs could be retained in corpus cavernosum, leading to their better therapeutic efficacy towards ED [59]. As mentioned earlier, the stimulatory effect of shock wave therapy (SWT) on ADSCs’ therapeutic efficacy seems noticeable. In a report, a combined treatment of ADSC and SWT significantly enhanced α-SMA content, neural nitric oxide synthase of dorsal penile nerve, endothelial nitric oxide synthase protein expression, and cyclic guanosine monophosphate level [60]. At the individual level, ADSCs improved the expression of β-III tubulin; whereas, low energy SWT improved angiogenesis in corpus cavernosum and recovered erectile function in a rat model of post-prostatectomy. A recent interesting study indicated that when compared to free ADSC suspension of the same dose, the intracavernous transplantation of size-specific ADSC-based spheroids showed better improvement in erectile functions [61]. However, higher doses of free ADSCs have been suggested to achieve similar efficacy as these small-sized cells could escape from the sponge-like corpus cavernosum after intracavernous injection.

### 3.3. Bone Marrow-Derived Mesenchymal Stem Cells 

Bone marrow-derived (BM)-MSCs are another considerable source for developing regenerative therapies. The intracavernous injection of BMSCs expressing receptor for p75 nerve growth factor (p75dMSCs) has resulted in superior ICP/MAP in Sprague-Dawley rats, possibly due to excess release of β-FGF, β-NGF, VEGF, and IGF-1 [62]. In an important report, both the intracavernous (IC) and intraperitoneal (IP) injection of BMSCs improved neurofilament, endothelial cell, and muscle content in cavernous tissues [63]. The therapeutic efficacy of MSCs could also be achieved by genetically modifying them through adenovirus-mediated transfection, leading to enhanced levels of endothelial nitric oxide synthase (eNOS) and cGMP, resulting in considerable improvement in erectile function [64]. In a recent pre-clinical rat model of CNI, the overexpressing miRNA-145 BMSCs revealed an improved ICP/MAP and smooth muscle content in penile tissues with suppressed levels of collagen type 1, matrix metalloproteinase 2 (MMP2), and phospho-Smad2 (p-Smad2) [65]. Compared to normal MSCs, the overexpressing stromal derived factor-1 (SDF-1)-engineered MSC treatment resulted in higher ICP (indicator of erectile function), smooth muscle content, vascular endothelial growth factor (VEGF), and basic fibroblast growth factor (bFGF) and lowered levels of the apoptosis factors Bcl2-associated x (Bax) and caspase-3 [66]. In a recent study by Fang et al., the combined BMSCs and human endothelial progenitor cells (hEPC) also elevated the endothelial and smooth muscle contents of corpus cavernosum, decreased caspase-3 expression, and increased the expression of penile neuronal nitric oxide synthase, leading to the restored neural component of the major pelvic ganglia in rats with CNI [67]. A phase I clinical trial using autologous BMSCs overcame ED in diabetic patients by improving erectile function without any significant adverse reactions and pain [68]. Further, a pooled result of stem cell therapies from three randomized clinical trials, i.e. transendocardial injection in patients with ischemic cardiomyopathy (poseidon), transendocardial mesenchymal stem cells and mononuclear bone marrow cells for ischemic cardiomyopathy (tac-hft), and dose comparison study of allogeneic mesenchymal stem cells in patients with ischemic cardiomyopathy (TRIDENT), revealed that in men with ischemic cardiomyopathy and ED, the delivery of high dose and autologous stem cell therapy into the myocardium may have a possible impact on erectile function [69]. Despite the improvements in endothelial function noticed in men, a translatable therapeutic success in erectile function has not appeared. Moreover, this study focused mainly on exploratory phase I/II trials and the male population, and a therapeutic benefit was not clearly defined.

### 3.4. Induced Pluripotent and Other Stem Cell-Based Approaches for ED

To avoid the ethical concern associated with embryonic stem cells (ESCs), induced pluripotent stem cells (iPSCs) are considered as a viable therapeutic alternative source. iPSCs are produced by inducing the expression of four main genes, *Oct3/4*, *Sox2*, *Klf4* and *c-Myc*, in somatic cells [70]. Similar to ESCs, iPSCs exhibit potential to differentiate into all three germ cells, i.e., ectoderm, mesoderm, and endoderm, compared to MSCs, which differentiate into limited cell lines [71]. iPSCs may considerably increase ICP/MAP, eNOS, and S100β content in MPG, leading to restored cavernous nerve integrity [72]. These regenerative effects could be ascribed to the anti-apoptotic activity and paracrine effect of iPSCs’ secretome. Besides, other sources of stem cells such as umbilical cord, skeletal muscles, penile tissues, and skin have been explored to develop regenerative treatment for ED [73]. Neural embryonic stem cells (NES) have also been administered in corpus cavernosal tissues and MPG to regenerate cavernosal nerve from crush injury [74]. These cells were able to improve ICP significantly and increase NOS-containing nerve fibers with enhanced neurofilament content. The proposed mechanism underlying this therapy is associated with the release of substrates from NES for axonal extension, control in demyelination, and release of growth factors. Nonetheless, iPSCs are a viable choice for regenerative therapies due to their pluripotency, yet the risks of genetic change, tumor formation, and epigenetic memory limit their clinical use [35].

Besides, ED patients exhibit a reduced number of circulating endothelial progenitor cells (EPCs), which is associated with poor endothelial function, possibly as a result of underlying low-grade inflammation [75,76]. Therefore, attempts have been made to administer exogenous EPCs to suppress ED characteristics. Reports have shown that preclinical intracavernous injection of EPCs in a bilateral cavernous nerve injury (BCNI) rat model improved smooth muscle, ICP, and eNOS content, which resulted in ED recovery [77]. Further, genetic modifications of EPCs have also been found effective in treating ED. In a study, the rat EPCs overexpressed with human telomerase reverse transcriptase restored erectile function in diabetic-induced ED rats by resulting in more secreted growth factors, greater smooth muscle content, and retaining stem cells in penile tissues [78]. Similarly, the administration of VEGF165-transfected EPCs into corpora cavernosa of rats with diabetic ED restored erectile function due to their enhanced survival, differentiation into endothelial cells, and integration into neovascularization sites [79]. Apart from this evidence, supplementation of nutraceuticals may also increase circulating levels of EPCs, which would possibly improve erectile function by inhibition of inflammation [75]. Thus, it is evident that EPCs are also potent candidates to restore erectile functions, yet the lack of sufficient preclinical and clinical evidence restricts their potential therapeutic use.

## 4. Cell-Free Regenerative Treatment

Though the mechanism of action of stem cell therapy is not well understood, their released factors like extracellular vesicles (EVs) have been attributed to exert a paracrine effect on injured tissues and have been explored for their efficacy towards ED.

### Stem Cell-Derived EVs in ED Treatment

The extracellular derivatives of stem cells seem to be effective in regenerative therapies [80,81]. Exosomes derived from ADSCs (ADSC-Exo) and BMSCs (BMSC-Exo) of 30–100 nm in size have been demonstrated to restore erectile functions of bilateral CNI rats by increasing levels of nNOS, neurofilaments, regenerated endothelial cells, nNOS-positive nerve, and MPG in penile dorsal nerve, resulting in improved ICP and SMC/collagen in corpus cavernosum [82]. In diabetes-induced ED rats, the EV derived from human urine stem cells (hUDSCs-EV) led to an increased miRNA-mediated angiogenesis, overexpression of nNOS and eNOS, and improvement in smooth muscle cells/collagen and ICP/MAP, indicating functional recovery [81]. Besides, the microRNAs (miRNAs) are integral parts of stem cells’ exosomes and paracrinally contribute to regenerative activities [83]. Several studies have also reported the anti-apoptosis and angiogenesis promoting roles of miRNAs such as miR-21, miR-124, and miR-31 [84,85,86]. In a seminal study, the transplanted UDSC-EVs enriched with miRNA families (miR-21-5p, let-7 family, miR-10 family, miR-30 family, and miR-148a-3p) in corpus cavernosum resulted in improved ICP and ICP/MAP ratio along with increased expression levels of CD31, eNOS, phospho-eNOS, nNOS, and the ratio of smooth muscle to collagen in in diabetic ED rats [81]. The therapeutic use of EVs overcame the risk of cellular contamination and unregulated cellular division, and therefore imparted multiple synergistic effects, which may be manipulated to enhance their clinical impact [87,88]. The secretion and composition of EVs vary according to the culture conditions and environmental conditions provided to stem cells. Though stem cell-derived EVs are gaining attention for regenerative therapy, the lack of established protocols for EV production with uniform characteristics, safe doses, and procedures to characterize EVs limit their clinical application [89]. Hence, prior to conducting clinical trials and therapy, the purity, stability, safety, and potency of EVs must be ensured [90].

## 5. Platelet-Derived Biomaterials in ED Treatment

**Figure 5:**
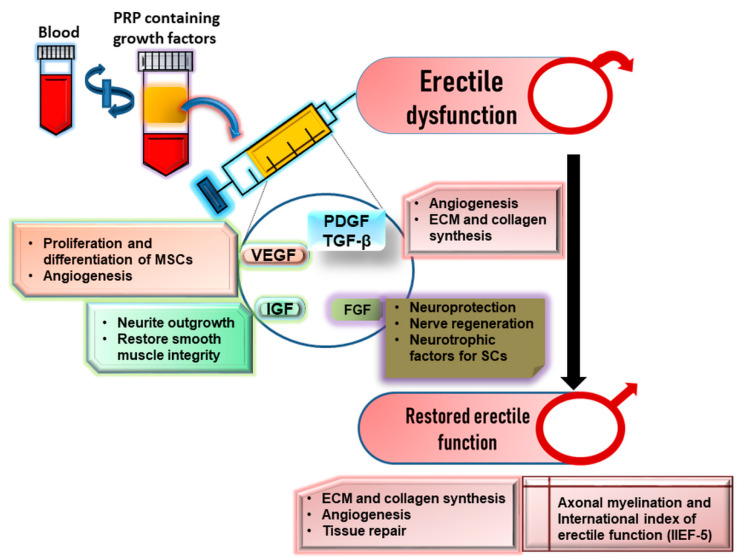
Platelet-rich plasma (PRP) as a source of platelet-derived biomaterials for ED therapy. Blood-derived PRP is highly rich in growth factors such as VEGF, IGF-1, FGF, PDGF, and TGF-β and plays a critical role in the proliferation and differentiation of MSCs, angiogenesis, blood flow regulation, neuro protection, nerve regeneration, axonal myelination, ECM, and collagen synthesis, resulting in an improved index of erectile function (IIEF-5). IGF: insulin-like growth factor, VEGF: vascular endothelial growth factor, FGF: fibroblast growth factor, PDGF: platelet-derived growth factor, TGF-β: transforming growth factor beta.

Blood-derived platelet-rich plasma (PRP) has been considered as a potential regenerative material to overcome various disorders including musculoskeletal and wound disorders [91]. PRP is harvested through centrifuging blood and needs to be activated for release of contained therapeutic biomaterials to exhibit a therapeutic regenerative effect. However, the application of PRP in directing the regeneration of injured tissues needs to be optimized as per the need and type of damaged tissues for repair (Figure 5) [92].

### 5.1. Synthesizing Regenerative PRP

While synthesizing PRP, the harvested blood sample is centrifuged, which is later separated into three layers, i.e., plasma, buffy coat rich in platelet-derived biomaterials, and red blood cell pellet [93]. The PRP contains around 1,000,000 platelets/μL in 5 mL of plasma, and this concentration has widely been reported for its therapeutic efficacy [94]. Platelets are actively involved in homeostasis, tissue regeneration, vascularization, and wound healing through their releasates, which are characterized as adhesive proteins, angiogenic factors, growth factors, chemokines, clotting factors and inhibitors, integral membrane proteins, immune mediators, and other active molecules [95]. These factors mainly include platelet-derived growth factor (PDGF), transforming growth factor-β (TGF-β), insulin-like growth factor (IGF), fibroblast growth factor-2 (FGF-2), vascular endothelial growth factor (VEGF), etc., which are mainly released from α, δ, and λ granules of activated platelets [96,97,98,99] (Table 1). Besides, other biological factors and cytokines such as connective tissue growth factor (CTGF), basic fibroblast growth factor (bFGF), platelet factor-4 (PF-4), keratinocyte growth factor (KGF), TNF-α (tumor necrosis factor-α), stromal-derived factor 12 (SDF-12), bone morphogenetic proteins (BMP)-3, BMP-4, BMP-5), and interleukin-2 (IL-2) and IL-8 [97,100] have also been reported to mediate the signaling pathway associated with repair and regeneration of damaged tissues actively. The growth factors promote cellular interaction and differentiation, and proliferation of localized mesenchymal and epithelial cells, leading to connective tissue regeneration [101,102,103]. A clinical study evidenced that PRP containing PDGF in the largest amount facilitated recovery in erectile function when injected in corpus cavernosum of rat with bilateral cavernous injury [104]. Initially, the concentration gradients of TGF-β and PDGF promoted wound healing and also triggered the release of other growth factors and the production of extracellular matrix (ECM) [105,106]. PDGF and TGF-β further promoted collagen synthesis, angiogenesis, chemotaxis, and protection of collagen breakdown, leading to tissue regeneration [99,107,108]. While deducing the mechanistic basis, Ingman et al. revealed the contribution of TGF-β1 to ED in mutant mice, where the absence of TGF-β1 altered the structural integrity and compliance of penile skin and tissues, resulting in ED [109]. The key functions of VEGF include angiogenesis, whereas FGF regulates the mitogenic activities of stem cell and also promotes the angiogenic activity of VEGF [110,111]. Of note, the significant decrease in IGF-1 is considered as an indicator of ED due to its correlation with endothelial dysfunction [112]. Therefore, IGF-1-enriched PRP could be employed for repairing and regenerating cavernous nerve for the treatment of ED by enhancing neurite outgrowth [113]. Delivery of recombinant VEGF such as adeno-associated virus-mediated VEGF gene therapy may also prevent venogenic ED through regenerating smooth muscle and nerve cells [114,115]. Further, an enhanced injury healing has been evidenced through leukocyte-rich PRP showing an increased chemotaxis and chemokinesis of MSCs compared to PDGF alone [116]. In a seminal study, ADSCs expressing VEGF improved erectile function in diabetic ED rats though raising ICP and ICP/MAP, which was associated with an increased number of smooth muscle cells and pericytes along with endothelial cells [117].

PRP is activated to release growth factors by using stimulants such as thrombin, calcium chloride, and collagen; however, the exact concentration and procedure for PRP activation is not well established [118]. Cryopreservation and freeze drying are also effective approaches to enable PRP storage with a maintained baseline level of growth factors and their biological activities with efficacy for extended periods. Cryopreservation and freeze drying could also maintain growth factors for longer time periods [119,120]. Freeze drying has been shown to maintain platelet counts even after four weeks and effectively induced osteoblast proliferation [121]. Beside, PRP and platelet-rich fibrin (PRF) polymerize rapidly, produce a more solid-like gel structure, and have been recommended to use in wound healing, ulcers, and sports-related injury [122]. The fibrin network could stabilize the solid gel network and control the slow release of growth factors [123]. Nonetheless, PRF were found to be safe and effective at providing therapeutic relief in urological disorders [124]. However, to establish the role of PRP in ED fully, extensive clinical studies are required to optimize the therapeutic protocol and hence the efficacy and safety.

### 5.2. Clinical Efficacy of PRP in ED

Attempts have been made to improve the clinical impact of PRP through optimizing and activating PRP. Apart from conventionally employed CaCl_2_, the cold shock treatment to PRP in the presence of anticoagulant and chitosan may increase the stable release of PRP-contained growth factors [104]. A clinical study revealed that autologous PRP may recover erectile function through the release of its active biomaterials, such as PDGF-AA, PDGF-BB, VEGF, VEGF-D, FGF, and FGF-acid, the concentration of which could be enhanced by freezing/thawing [125]. This may be attributed to endogenous NO enhanced VEGF synthesis, which supports endothelial regeneration via angiogenesis [126]. Interestingly, the synergistic effect of PRP and low-intensity shock waves (LI-SW) has been demonstrated to ameliorate ED in patients [127]. Even though the peripheral nervous system possesses the ability to restore the functional activity of damaged nerves, their complete or optimal recovery is not achieved. Therefore, PRP may be applied as a filler or scaffolds to bridge nerve conduits, as PRP rich in fibrin matrix controls the sustained release of neurotrophic growth factors, which regulate angiogenesis, inflammation, apoptosis, fibrosis, and muscle atrophy, thereby promoting rapid regeneration of the injured peripheral nerve gap [128]. This therapeutic functional recovery is also associated with PRP’s potential to maintain balance between the synthesis of collagen I, III, and IV [129]. To prevent the concern of early wash out, PRP could be converted to platelet-rich fibrin (PRF) rich in fibrinogen, fibronectin, platelets, leukocytes, and other growth factors [124]. This approach restricts the rapid removal of platelet-derived biomaterials from cavernous nerve and limits the risk of reduced blood flow. It has been reported that S1P and RhoA/ROCK1 signaling might be involved in corporal fibrosis with loss of smooth muscles during CN injury [130]. In crushed bilateral cavernous nerve, the administered PRP has been evidenced to improve ICP, preserve myelinated axons, and reduce the apoptotic index [131]. This evidence implies that platelet-derived biomaterials could inhibit S1P and RhoA/ROCK1 signaling and suppress nerve fibrosis. The other suggested underlying pathogenic mechanism of cavernous fibrosis involves pathways like RhoA-ROCK1-LIMK2-cofilin, Smad, Sonic hedgehog signaling, angiotensin-II-Smad, HDAC4-TGF-β1-Smad signaling, p42-44, and mitogen-activated protein kinase [132], the inhibition of which could protect erectile function through suppressing corporal fibrosis [133]. Under diabetic conditions, over-activated TGF-β1 and its Smad and non-Smad pathways may increase collagen content, resulting in structural alterations in corpus cavernosum through cavernosal fibrosis and severe corporal veno-occlusive dysfunction [134]. These pathogenic characteristics could be lessened by platelet-derived biomaterials, such as TGF-β2, IGF-I, and VEGF, which may participate in neural regeneration and upregulation of nNOS. Since neuronal cells express PDGF receptors, PDGF-β has been demonstrated to be a mitogen for Schwann cells with trophic activity on neurons [135]. After peripheral nerve injury, the presence of enhanced levels of PDGF-β in peripheral neurons indicates its key role in peripheral nerve regeneration [136].

### 5.3. Synergistic PRP and Stem Cell Therapies Against ED

The potential of PRP in the regeneration of crushed cavernosal nerve to restore erectile function opens an approach to establish a symbiotic therapy with stem cells. PRP enriched in growth and neurotropic factors promotes the proliferation and differentiation of stem cells towards the neural lineage [137]. In a rat model of spinal cord injury, a combined treatment of PRP and BDNF-overexpressing BMSCs showed an increased expression of glial fibrillary acidic protein and promoted the migration of astrocytes into the transplants, as well as the axonal re-myelination [137]. A single center pilot study showed that only PRP or combined with ADSC injections could positively contribute to ED treatment, which was revealed through a significantly improved International Index of Erectile Function (IIEF-5) [138]. Similarly, the therapeutic additive role of PRP on neural-induced human mesenchymal stem cells was evidenced in a guinea pig model of facial nerve axotomy injury, where significant improvements in axon count and myelination of axon fiber were exhibited, resulting in functional improvement in facial movements [139]. The PRP fibrin scaffold releases growth factors, neurotrophic and mitogenic factors, and cytokine/chemokines at the target injury site [140], which exert anti-apoptotic and neuro-protective impacts on stem cells. These activities of the PRP-derived scaffold showed clear superiority when supplemented with BDGF, leading to human BMSC survival and their differentiation towards a neural phenotype [141,142]. In an exciting study, an intranasally-administered PRGF (plasma rich in growth factor), Endoret, in an Alzheimer’s disease mouse model, decreased brain β-amyloid deposition, indicating its neuro-protective and anti-apoptotic effect [143]. PRP when mixed with Schwann cell-like cells along with poly(lactic-co-glycolic acid) conduits demonstrated a greater number of neurons in dorsal root ganglion and spinal cord anterior horn, implying a considerable recovery of the injury of sciatic nerves in rabbits [143]. This body of evidence clearly implies that compared to individual treatment, the combinatorial therapeutic approaches of PRP and stem cells possess better potential for nerve regeneration, and hence could be further explored as alternative remedies for ED.

## 6. Discussion and Future Prospects

The current treatment options to overcome ED have enormously improved the sexual life of males and therefore play a crucial role in restoring confidence and relationships. However, these treatments could only provide limited recovery from CNI-induced ED. The demand for highly efficacious and long-lasting therapeutic options has prompted the scientific community to explore the potential of regenerative medicine. The current surge in scientific, preclinical, and clinical studies in regenerative therapies including stem cells and PRP has not only increased the therapeutic expectations, but also uncovered the scientific rationale behind them. Both the stem cell, as well as PRP-based therapies have imparted nerve cell regeneration through secretion of growth and nerve factors and anti-apoptotic activities. The pre-clinical studies have shown significant improvement in terms of restoring erectile functions through cell-based therapies in ED animal models, which could be improved through LI-ESWT by increasing cell viability, proliferation, and multilineage differentiation [144]. A recent study showed an improved therapeutic efficacy of Schwann cells with overexpressing GDNF in CNI-induced ED rats [145]. Thus, the overexpression of relevant growth factors may be targeted to promote rapid stem cell-mediated regeneration of damaged cavernosal nerves. Moreover, the stem cells in combination with non-surgical and pharmacological agents might prove to be a crucial therapeutic strategy for ED.

Similar to stem cells, PRP has also been widely studied for its regenerative potential, which is ascribed to its capability to secrete growth factors, cytokines, and ECM, thereby promoting migration, proliferation, stabilization, and differentiation of endothelial, fibroblast, and stem cells [146]. Therefore, the concentrations of platelet, cytokine, and growth factors need to be well established to achieve the optimal therapeutic impact of PRP [147]. Further, regenerative potential could be enhanced through combining both stem cells and PRP. However, to date, the studies on PRP and stem cells remain limited to establishing the clinical efficacy and safety for CNI-induced ED. Hence, extensive, multicenter, and large preclinical and clinical studies are required for the acceptance of these regenerative therapeutics.

## Figures and Tables

**Table 1:** PRP-contained growth factors and their functional role in regenerative therapy. BMSCs: bone marrow stem cells, ECM: extracellular matrix, PRP: platelet-rich plasma.

PRP growth Factors	Regenerative Roles
Platelet-derived growth factor (PDGF)	Angiogenesis, ECM and collagen synthesis
Transforming growth factor-β (TGF-β)	Angiogenesis, immunomodulation, ECM and collagen synthesis
Insulin-like growth factor (IGF)	Neurite growth, restoration of smooth muscle cells integrity
Fibroblast growth factor-2 (FGF-2)	Neuroprotection, nerve regeneration, neurotrophic factor for stem cells
Vascular endothelial growth factor (VEGF)	Angiogenesis, proliferation and migration of endothelial and stem cells
Connective tissue growth factor (CTGF)	Proliferation, migration, and targeting of mesenchymal stem cells
Basic fibroblast growth factor (bFGF)	Promotes cell proliferation
Platelet factor-4 (PF-4)	Promotes platelet localized aggregation
Keratinocyte growth factor (KGF)	Promotes wound healing and secretion of growth factor
Tumor necrosis factor-α (TNF-α)	Immunomodulation and recovery in muscle and wound injury
Stromal-derived factor-1 (SDF-1)	Recruitment of BMSCs and endothelial progenitor cells at the injured site
Bone morphogenetic proteins (BMP): BMP-3, BMP-4, and BMP-5	Promotes synthesis of ECM
Interleukins (IL): IL-2 and IL-8	Immunomodulation and recovery in muscle and wound injury
